# A scoping review protocol to elucidate outcomes following abiraterone versus enzalutamide for prostate cancer

**DOI:** 10.1371/journal.pone.0273826

**Published:** 2022-08-29

**Authors:** Yash B. Shah, Amy L. Shaver, William Kevin Kelly, Grace Lu-Yao

**Affiliations:** 1 Sidney Kimmel Medical College at Thomas Jefferson University, Philadelphia, PA, United States of America; 2 Department of Medical Oncology, Sidney Kimmel Cancer Center at Jefferson, Philadelphia, PA, United States of America; 3 Jefferson College of Population Health, Philadelphia, PA, United States of America; Xiamen University - Malaysia Campus: Xiamen University - Malaysia, MALAYSIA

## Abstract

**Introduction:**

Abiraterone acetate and enzalutamide are commonly employed in prostate cancer therapy in an interchangeable manner. These drugs are highly efficacious in androgen antagonism to improve patient outcomes, but they also carry noteworthy risk of adverse effects. Common toxicities vary amongst the two drugs and may have differential interactions with patient co-morbidities, but these patterns are unclear as co-morbidities typically serve as exclusion criteria in clinical trials. Hence, there is no existing guidance on how clinicians may tailor treatment based on patient-specific factors. Analysis of differential patient outcomes between these two drugs can inform future systematic reviews, new clinical studies, and clinical decision making.

**Method and analysis:**

The framework for this methodology was informed by the Joanna Briggs Institute methodology for scoping reviews. Title and abstract screening will be performed by two independent researchers to create an initial study inventory. This will be followed by full-text screening for study inclusion. Population-based studies describing patient outcomes, common toxicities, and associations with patient co-morbidities following abiraterone or enzalutamide therapy will be included. After data is extracted, it will be summarized for presentation.

**Ethics and dissemination:**

The findings of this scoping review will be published in a peer-reviewed journal. The results will be used to inform future studies on patient-specific factors informing treatment choice between abiraterone and enzalutamide for castration-resistant prostate cancer. All data are from published openly accessible sources, and therefore, no ethical clearance is necessary. The protocol is also registered at https://doi.org/10.6084/m9.figshare.19149227.

## Introduction

Abiraterone acetate, an androgen biosynthesis inhibitor, and enzalutamide, the first-approved androgen receptor signaling inhibitor, are androgen deprivation therapies (ADTs) widely used as mainstay therapies for prostate cancer (PCa), particularly in metastatic disease. PCa is the most commonly diagnosed cancer in men, accounting for approximately 20% of new cancer cases [1, 2]. Although the survival rate for locoregional disease approaches 99%, that of advanced and metastatic cancers is markedly lower, making ADT outcomes crucial to urologic oncology [1]. However, treatment outcomes for real-world patients following abiraterone or enzalutamide have only been compared in small retrospective cohort analyses, which are limited in their applicability due to various biases [2].

Nonetheless, such studies have demonstrated likely associations between various metabolic, cardiovascular, neurological, and other co-morbidities with treatment toxicities and outcomes [3, 4]. For instance, cardiovascular disease is the most common co-morbidity and cause of death in prostate cancer patients, and its incidence is higher in these patients compared to the general population, making treatment evaluation markedly germane to this cohort [1].

Adverse effects are commonly seen with ADTs and largely vary between drugs. For instance, abiraterone has been shown to significantly increase cardiac risk while enzalutamide mounts hypertension [4–9]; however, these differential toxicities of abiraterone and enzalutamide, and particularly their interactions with pre-existing patient conditions, have not been fully elucidated, and clinicians continue to prescribe these drugs interchangeably.

Unfortunately, ADT clinical trials frequently exclude patients with co-morbidities, limiting the generalizability of their findings to the broader population [7]. By understanding real-world patient outcomes based on drug-associated toxicities and patient co-morbidity patterns, clinicians can perform an informed risk assessment to guide treatment choice and properly address toxicities should they occur following treatment administration.

Against this backdrop, the present scoping review will aim to describe the differential outcomes and adverse effects of abiraterone acetate and enzalutamide for PCa patients in the general population. This study can identify gaps in current utilization of ADTs, inform future clinical studies or systematic reviews, and ultimately inform patient risk assessment to guide treatment choice or toxicity prevention tools.

### Review question

What are the differential toxicities and outcomes of abiraterone acetate versus enzalutamide therapy for prostate cancer, and how do these relate to patient risk factors?

## Methods

### Protocol design

This scoping review follows the framework outlined by the Joanna Briggs Institute Manual for Evidence Synthesis (JBIMES), incorporating protocols established by Arksey and O’Malley along with revisions from Levac et al and Peters et al [10–14]. This review will include the following six steps: defining the research question; identifying relevant studies; study selection; charting the data; collating, summarizing, and reporting the results; and consultation. Findings will be reported according to the Preferred Reporting Items for Systematic Reviews and Meta-Analyses (PRISMA) guidelines utilizing the extension for scoping reviews (PRISMA-ScR) [15]. The PRISMA-ScR checklist is attached (S1 Checklist). This protocol has been registered at https://doi.org/10.6084/m9.figshare.19149227 [16].

### Identifying relevant studies

The review will primarily evaluate outcomes utilizing data regarding drug-associated toxicities, mortality, hospitalizations, and patient co-morbidities. Data on monitoring or mitigating drug-induced toxicities will also be gathered. Outcomes have been purposefully left broad to capture as much information as possible. All outcome data will be categorized in the “collating, summarizing, and reporting the results” stage.

### Search strategy

The search strategy for this scoping review is informed by prior research in prostate cancer therapy, as well as recommendations by Tawfik et al to adapt searches to the database being utilized [17]. An experienced search librarian was also consulted. We will conduct a search of PubMed, Cochrane Library, CINAHL, and Scopus.

We will conduct a search using the following keywords: “prostate cancer”, “prostatic neoplasms”, “abiraterone acetate”, “enzalutamide”, “toxicities”, “outcomes”, and associated MeSH terms. These terms will be combined with the Boolean operators “AND” and “OR”.

The initial search will be in PubMed. A similar search will be used for Cochrane Library and CINAHL, which also utilize MeSH terms. Only keywords will be used for Scopus.

The search string for the PubMed database is as follows:

("Prostatic Neoplasms"[MeSH Terms] OR "Prostatic Neoplasms"[All Fields] OR "prostate cancer"[All Fields]) AND ("Abiraterone Acetate"[MeSH Terms] OR "Abiraterone Acetate"[All Fields]) AND ("enzalutamide"[All Fields] OR "Androgen signaling inhibitor"[All Fields]) AND ("Drug-Related Side Effects and Adverse Reactions"[MeSH Terms] OR "Treatment Outcome"[MeSH Terms] OR "Hospitalization"[MeSH Terms] OR "Mortality"[MeSH Terms] OR "Comorbidity"[Mesh]).

### Types of participants

This scoping review will only include patients being treated with abiraterone acetate or enzalutamide for PCa.

### Concept

This review will focus on full-length peer-reviewed publications that elucidate and compare the observed outcomes and toxicities of abiraterone or enzalutamide and their possible associations with patient co-morbidities.

### Context

This review will focus on population-based studies from institutional and community care settings.

### Types of sources

All peer-reviewed publications through January 31, 2022.

### Exclusion criteria

The search will be restricted to articles and reports published in English. Free-standing abstracts, opinion pieces, and letters to the editor will be excluded. Studies investigating only one of either abiraterone or enzalutamide will be excluded. If further information is required, we will contact authors of the publications as appropriate.

### Study selection

Studies identified by the above search strategy which satisfy the initial inclusion criteria will be considered for title and abstract screening. The search strategy will be adapted for other databases as required. The reference lists of all included articles will be searched for additional studies. As required by good practice, the completed strings for each database will be included in the published scoping review.

Endnote 20 (Clarivate, Philadelphia, PA) will be employed for imported reference management and duplication removal. The title, abstracts, and keywords will be screened by two independent reviewers to determine whether they satisfy the inclusion criteria (S1 File). Articles satisfying initial screening will undergo full-text screening by two independent researchers. Disagreements of study eligibility will be resolved through discussion with a senior member of the research team.

### Charting the data

#### Extraction of the results

Three members of the research team will conduct data extraction. From each article, the following information will be extracted: author, year of publication, title, drug, study type/design, study population, primary objective(s), and outcome(s)/summary (S2 File).

#### Patient and public involvement

This research will be done without patients or public involvement. Patients are neither invited to comment on study design nor consulted to develop patient-relevant outcomes nor to interpret nor disseminate the results.

### Collating, summarizing, and reporting the results

Search results will be presented in a PRISMA flowchart and an appended PRISMA-Scr checklist (S1 Checklist). The extracted data will be presented under the following headings: author, year of publication, study type, study population, primary objective(s), and outcome(s).

A full summary of evidence, including an overview of concepts and types of evidence available, as well as a discussion of limitations and study conclusions, will follow. The research team will identify gaps in the literature and highlight implications for future research.

### Stage 6: Consultation

This protocol has purposefully included researchers from multiple disciplines (pharmacy, medicine, and epidemiology) within the research team. The diversity of the group brings unique views and broad experience to the literature analysis. At the end of the study, a final consultation will allow researchers to insert the context of clinical practice knowledge to the study results.

### Ethics and dissemination

As indicated earlier, the scoping review is based upon openly accessible published material and is therefore not subject to an ethical review board. The findings of this scoping review will be published in a peer-reviewed journal. The results will be used to inform future studies on patient-specific factors influencing risk assessment and treatment choice for abiraterone and enzalutamide.

## Conclusions

Prostate cancer is one of the most common oncologic diagnoses in men, and prognosis for advanced disease can be greatly improved. Scoping reviews allow a systematic approach to surveying and mapping existing research to better understand a domain of interest. This proposed study will allow urologic oncology to better understand the utility and outcomes of two key prostate cancer drugs- abiraterone acetate and enzalutamide- in the treatment of advanced prostate cancer, ultimately improving patient experiences and healthcare quality.

## Supporting information

S1 Checklist Preferred Reporting Items for Systematic reviews and Meta-Analyses extension for Scoping Reviews (PRISMA-ScR) checklist. (DOCX)

S1 File Inclusion assessment form. (DOCX)

S2 File Extraction/charting form. (DOCX)
