# Supplementary material for: A scoping review protocol to elucidate outcomes following abiraterone versus enzalutamide for prostate cancer
Source: PLoS One. 2022 Aug 29;17(8):e0273826. doi: 10.1371/journal.pone.0273826 (PMC9423609; doi:10.1371/journal.pone.0273826)
Supplement: S1 File — (DOCX) [file pone.0273826.s002.docx]

**Inclusion Assessment Form**

Outcomes Following Abiraterone versus Enzalutamide for Prostate Cancer: A Scoping Review

| Reviewer: | Date of Review: |
| --- | --- |
| Reference (Author, Year, Title): | |
|  | |

Instructions: Complete the form on each study. If the final decision is “no”, exclude that study.

**CRITERIA**

|  |  |  |  | |  | | **Yes** | | **No** | **Unsure** |
| --- | --- | --- | --- | --- | --- | --- | --- | --- | --- | --- |
| 1. Study Publication | | | |  | |  | |  |  |  |
|  | Is the study a full manuscript? | | | | | | [ ] | | [ ] | [ ] |
|  | Was the study published by January 31, 2022? | | | | | | [ ] | | [ ] | [ ] |
|  | Was the study published in English? | | | | | | [ ] | | [ ] | [ ] |
| 2. Study Population | | | |  | |  | |  |  |  |
|  | Did the study include patients diagnosed with prostate cancer? | | | | | | [ ] | | [ ] | [ ] |
| 3. Study Exposure | | | |  | |  | |  |  |  |
|  | Were patients receiving treatment with either abiraterone or enzalutamide? | | | | | | [ ] | | [ ] | [ ] |
|  | Did patients have exclusion criteria based on co-morbidities? | | | | | | [ ] | | [ ] | [ ] |
| 4. Study Outcomes | | | |  | |  | |  |  |  |
|  | Did the study report on at least one patient outcome?  i.e. the study is not an opinion, editorial, summary of function, etc. only | | | | | | [ ] | | [ ] | [ ] |
|  | Are there at least two separate treatment groups (for abiraterone and enzalutamide) which are compared? | | | | | | [ ] | | [ ] | [ ] |

**DECISION OF REVIEWER**

|  |  |  |  |  | **Yes** | **No** | **Unsure** |
| --- | --- | --- | --- | --- | --- | --- | --- |
| 1. Is this study potentially relevant for this review? | | | | | [ ] | [ ] | [ ] |

**CONSENSUS**

Second review [ ] Include

[ ] Exclude

[ ] Disagree

Final consensus [ ] Include

[ ] Exclude

[ ] Disagree
