# Supplementary material for: A scoping review protocol to elucidate outcomes following abiraterone versus enzalutamide for prostate cancer
Source: PLoS One. 2022 Aug 29;17(8):e0273826. doi: 10.1371/journal.pone.0273826 (PMC9423609; doi:10.1371/journal.pone.0273826)
Supplement: S2 File — (DOCX) [file pone.0273826.s003.docx]

**Extraction/Charting Form**

| Author(s)^*^ | Year | Title | Study type | Population | Treatment | Objective/ Outcomes | Context and Findings |
| --- | --- | --- | --- | --- | --- | --- | --- |
|  |  |  |  | Prostate cancer patients | Abiraterone vs. Enzalutamide | Measured outcomes including toxicities, survival, and hospital length of stay |  |
|  |  |  |  |  |  |  |  |
|  |  |  |  |  |  |  |  |
